# The Effect of Personality Traits on Patient Compliance With Clear Aligners

**DOI:** 10.7759/cureus.74922

**Published:** 2024-12-01

**Authors:** Salma H Ghoneim, Khaled S Afif

**Affiliations:** 1 Department of Orthodontics, King Abdulaziz University, Jeddah, SAU

**Keywords:** adherence, bfi-10, big five inventory, clear aligner treatment, demographic factors, orthodontics, orthodontic treatment compliance, patient compliance, personality and treatment compliance, personality traits

## Abstract

Introduction: Patient compliance is crucial for achieving optimal outcomes in clear aligner (CA) therapy. Compliance may be influenced by various factors, including demographics, level of education, doctor-patient interaction, and personality traits based on the Big Five Inventory (BFI), which assesses openness, conscientiousness, extraversion, agreeableness, and neuroticism. This study investigates the relationship between personality traits and compliance among CA patients.

Methods: A cross-sectional study was conducted with 67 participants aged 12-60 undergoing CA treatment in Jeddah, Saudi Arabia. Patients completed an online questionnaire that assessed compliance behavior and personality traits using the BFI-10 tool. Data were statistically analyzed using SPSS software, version 26 (IBM Corp., Armonk, NY), to examine correlations between personality traits, demographic factors, and adherence to CA therapy. Compliance was scored based on adherence to follow-up visits and aligner wear time.

Result: The study revealed that 34 participants (50.75%) demonstrated high adherence to the prescribed regimen. Males showed significantly higher compliance than females (p ≤ 0.05). Participants aged 12-34 exhibited the highest adherence rates (p ≤ 0.05), and those undergoing treatment for one year or less were also more compliant (p ≤ 0.05). Notably, satisfaction with one's smile did not significantly correlate with adherence (p > 0.05), contradicting the assumption that dissatisfaction with appearance drives better compliance. Furthermore, no significant association was identified between personality traits across any BFI domain and adherence.

Conclusion: Despite the hypothesis that personality traits influence patient compliance, this study did not find a significant correlation. These findings suggest that factors other than personality traits may be more critical to adhering to CA treatment. The results highlight the need for further research to explore additional variables that may impact patient compliance in orthodontic therapy.

## Introduction

Ensuring compliance with clear aligner (CA) therapy is crucial for attaining desired treatment outcomes. Patients are advised to wear aligners 22 hours a day [1,2]. Hence, the most significant factor in the effectiveness of CA treatment remains compliance during all phases of treatment [3]. Numerous variables, including socioeconomic and demographic factors, level of education, doctor-patient relationship, general treatment information, family history, regimen, comfort, the influence of the treatment provider, and parental supervision, impact the overall compliance of patients. According to Lim et al. [4], the main obstacles to wearing removable orthodontics were speech difficulties, discomfort, and forgetfulness. Furthermore, they found that employment status and age can affect patient compliance.

An individual's personality traits are another variable affecting compliance. Personality is considered an intrinsic factor that represents a distinctive feature of an individual, shaped by different behaviors, thoughts, and feelings. The Big Five Inventory (BFI) identifies five personality traits: openness, conscientiousness, extraversion, agreeableness, and neuroticism, each with distinct characteristics [5]. Initially consisting of 44 questions (BFI-44), the inventory also has a shorter version (BFI-10) with 10 questions to measure these traits. This brief form is considered a valid and reliable tool for assessing personality and can be completed in one minute [6]. The BFI has been translated into various languages, including Arabic, with proven reliability [7].

Many studies have explored the factors influencing compliance, including age, gender, employment status, and socioeconomic background [8,9]. Research consistently shows that personality traits also play a role in compliance across various fields [10,11]. Specifically, traits such as agreeableness and conscientiousness tend to have a positive association with compliance [12], whereas neuroticism and extraversion are often linked to lower compliance levels [13].

A study evaluated the relationship between personality traits and willingness to endure various orthodontic treatment procedures, such as mini-screw placement, avoiding hard or sticky food, or wearing retainers, headgear, elastics, and aligners. The findings revealed that three personality traits were associated with different orthodontic treatments; for example, neuroticism was negatively correlated with the willingness to avoid hard foods, while agreeableness was positively linked to the willingness to use aligners, elastics, retainers, and a Herbst appliance. Additionally, wearing a Herbst was associated with lower conscientiousness [14].

Another study discussed the effects of personality traits on the perception of esthetic procedures and how this might affect compliance with the regimen. They found that high levels of agreeableness and openness were linked to a positive perception of esthetic treatments, which affects the treatment [15]. This research holds significant value as it addresses an understudied area by exploring personality traits as predictors of compliance with CA therapy. While adherence is crucial for the success of orthodontic treatment, there is limited research globally and particularly none in Saudi Arabia or the broader Middle East, which examines the role of individual personality traits in influencing compliance in the dental field as it has been searched in other medical fields [16].

This study aims to bridge this gap by investigating how traits, including openness, conscientiousness, extraversion, agreeableness, and neuroticism, affect patient adherence. By utilizing tools such as the BFI-10 survey, practitioners can assess a patient's likelihood of compliance before treatment begins. This insight might enable orthodontists to adopt more personalized treatment strategies, improving outcomes and optimizing treatment efficiency. The findings have the potential to contribute to both global and regional orthodontic practices by offering a novel, evidence-based approach to identifying compliant patients for better patient selection for CA therapy.

## Materials and methods

Study design and participant selection

This cross-sectional study was conducted in Jeddah, Saudi Arabia. Patients using removable CA met the study's inclusion criteria. Patients using other types of orthodontic treatment and all surgical cases were excluded. A convenience sampling technique was employed, where participants were selected from multiple orthodontic clinics in Jeddah. Ethical approval was obtained from King Abdulaziz University's ethics committee, and informed consent was obtained from all participants.

Data collection, research instrument, and survey

Data were collected through an online questionnaire hosted on Google Forms. The questionnaire was embedded in a QR code and distributed in orthodontic clinics. The first page of the questionnaire contained an informed consent section, and participants who consented and met the inclusion criteria were directed to the second page containing the questionnaire.

Questionnaire design and testing

The data collection tool comprised two parts. The first was the BFI (BFI-10), which assesses personality traits. The second was a questionnaire developed by the research team, with input from three expert physicians, designed to evaluate patient compliance with CA treatment.

Pilot study

A pilot test was conducted with 10 participants to assess the reliability and clarity of the questionnaire. Participants were selected based on the inclusion criteria, and the questionnaire was administered twice two weeks apart to ensure temporal consistency. Following the pilot study, minor adjustments were made to improve question clarity.

Survey scoring

The compliance questionnaire consisted of seven questions. A score was given for each question, depending on the patient's answer. For these two questions (How often were you instructed to visit the doctor for CA follow-ups? and How often do you actually visit the doctor for follow-ups?), the scoring was given according to the patient's compliance with the instructions given by the orthodontist: if he followed the exact instruction (score = 3), if he followed the instruction approximately (score = 2), if he came in for follow-ups but different from what he was instructed (score = 1), and if the patient did not come for follow-ups or just came in when problems happened (score = 0). For these two questions (Do you wear your orthodontic CA at work? and Do you wear your orthodontic CA at school or college?), only one of the questions was scored according to whether the patient was at school age or labor age. The questionnaire and scoring system are illustrated in Appendixes 1, 2.

Sampling and sample size

The sample size formula was used to determine the number of participants needed, with a power of 85% and a significance level of 0.05. The calculation resulted in a required sample size of 63 participants, assuming a variance of 0.015-0.02 and an average proportion of 0.45-0.50. A convenient sampling technique was employed over a three-month period (from June to September 2024), inviting patients from different orthodontic clinics who met the eligibility criteria.

Statistical analyses

The statistical analysis comprised both descriptive and correlational statistics. Data were statistically analyzed using the SPSS software, version 26 (IBM Corp., Armonk, NY). To analyze the association between the variables, Fisher's exact test was applied to qualitative data expressed in numbers and percentages. Quantitative data were represented as mean and standard deviation, and nonparametric variables were analyzed using Mann-Whitney and Kruskal-Wallis tests. A p value of <0.05 was considered statistically significant. The average compliance score was 22.0 (±3.8) (min 11, max 26). Participants were categorized as low compliers if their overall score was ≤22 and high compliers if it was >22.

## Results

Demographic data are presented in Table 1, showing that the majority of the sample were females 49 (73.1%), while 39 (58.2%) held a bachelor's degree.

**Table 1:** Sociodemographic characteristics of study participants

Variable	n (%)
Age (years)	
12-24	23 (34.3)
25-34	26 (38.8)
35 or more	18 (26.9)
Gender	
Male	18 (26.9)
Female	49 (73.1)
Education level	
High school or less	17 (25.4)
Bachelor’s degree	39 (58.2)
Higher education	11 (16.4)

Almost half of the participants, 34 (50.7%), have been undergoing orthodontic CA treatment for more than a year, and the majority, 55 (82.1%), were instructed to visit the doctor for CA follow-ups every one to two months. Interestingly, 48 (71.6%) patients visited the doctor for follow-up every one to two months as instructed. Approximately 38 (56%) participants wore the orthodontic CA at school or college all or most of the time, but just roughly 14 (21%) wore it at work. The majority of participants 65 (97%) wore the CA while sleeping all or most of the time. More than half of the participants, 37 (55.2%), removed their orthodontic CA only one to three times per day, with the most common reason for removal being eating 67 (100%) and brushing their teeth 59 (88.1%).

The majority of participants, 38 (56.7%), were instructed by the doctor to wear the CA for 22 hours or more, with 36 (53.7%) wearing it for 20-22 hours. Most of them, 48 (71.6%), reported that they wore the CA for the number of hours prescribed by the doctor.

The most prevalent reason for not wearing it as instructed was that they did not want to remove it to eat in front of others 12 (63.2%), followed by the belief that their wearing time is adequate and effective six (31.6%), and speech impairment six (31.6%) (Table 2).

**Table 2:** Compliance questionnaire and answers (n = 67)

Variable	Response	n (%)
When did you first start wearing clear aligners?	Less than a month	6 (9.0)
	1-3 months	8 (11.9)
	4-6 months	9 (13.4)
	7-12 months	10 (14.9)
	More than a year	34 (50.7)
How often were you instructed to visit the doctor?	1-2 months	55 (82.1)
	3-6 months	10 (14.9)
	7 months or more	1 (1.5)
	When finished set	1 (1.5)
How often do you visit the doctor for follow-up?	1-2 months	48 (71.6)
	3-6 months	15 (22.4)
	7 months or more	2 (3.0)
	When finished set	1 (1.5)
	When there is a problem	1 (1.5)
Do you wear the aligner at work?	Always	8 (11.9)
	Most of the time	6 (9.0)
	Sometimes	3 (4.5)
	Rarely	2 (3.0)
	Never or not applicable	48 (71.6)
Do you wear the aligner at school or college?	Always	29 (43.3)
	Most of the time	9 (13.4)
	Sometimes	4 (6.0)
	Rarely	1 (1.5)
	Never or not applicable	24 (35.8)
Do you wear the aligner on weekends/off days?	Always	42 (62.7)
	Most of the time	14 (20.9)
	Sometimes	9 (13.4)
	Rarely	2 (3.0)
	Never or not applicable	0 (0)
Do you wear the aligner while sleeping?	Always	64 (95.5)
	Most of the time	1 (1.5)
	Sometimes	2 (3.0)
	Rarely	0 (0)
	Never or not applicable	0 (0)
How many times do you remove the aligner per day?	1-3 times	37 (55.2)
	4 times	21 (31.3)
	5 or more times	9 (13.4)
Why do you remove the aligner?	When I brush my teeth	59 (88.1)
	For eating	67 (100)
	Just to rest	1 (1.5)
	For drinks	33 (49.3)
	At work	4 (6.0)
	When I smoke	4 (6.0)
	At school or college	2 (3.0)
	When I talk to others	3 (4.5)
Doctor's recommended hours for wearing aligner	22 hours or more	38 (56.7)
	Most of the day	29 (43.3)
	12 hours	0 (0)
	Only while sleeping	0 (0)
	No instructions given	0 (0)
	Forgot instructions	0 (0)
Actual hours of wearing the aligner	20-22 hours	36 (53.7)
	16-19 hours	19 (28.4)
	9-15 hours	11 (16.4)
	8 hours or less	1 (1.5)
Do you wear the aligner as recommended?	Yes	48 (71.6)
	No	19 (28.4)
Why don’t you wear the aligner as recommended? (n = 19)	Don’t want to remove it in front of others	12 (63.2)
	Wearing time is enough/effective	6 (31.6)
	Affects speech	6 (31.6)
	Doesn’t fit properly	1 (5.3)
	Affects appearance	1 (5.3)
	Pain/discomfort	4 (21.1)
	Other	5 (26.3)
Do you like your smile?	Yes	62 (92.5)
	No	5 (7.5)
Do you trust your doctor?	Yes	67 (100)
	No	0 (0)

The average compliance score was 22.0 (±3.8) (min 11: max 26). Approximately 34 (50.75%) participants adhered to CA therapy. Table 3 shows the relationship between compliance with CA and participants' demographics, duration of orthodontic CA treatment, and BFI types. High compliers ranged between the ages of 12 and 34 (p ≤0.05). Males were more compliant than females (72.2% vs. 40.8%, respectively) (p ≤ 0.05). Furthermore, participants who started treatment for one year or less had higher compliance rates (p ≤ 0.05).

**Table 3:** Relationship between the compliance mean score and participants’ demographics, if they like their smile and BFI type Please note that the test used was the “Fisher exact test” ^*^Statistically significant (p < 0.05) CA: clear aligner; BFI: Big Five Inventory

Variable	Score range/response	Low compliance, n (%)	High compliance, n (%)	p value
Age (years)	12-24	9 (39.13)	14 (60.87)	0.027^*^
	25-34	11 (42.31)	15 (57.69)	
	35 or more	14 (77.78)	4 (22.22)	
Gender	Male	5 (27.78)	13 (72.22)	0.023^*^
	Female	29 (59.18)	20 (40.82)	
Education level	High school or less	10 (58.82)	7 (41.18)	0.132
	Bachelor’s degree	16 (41.03)	23 (58.97)	
	Higher education	8 (72.73)	3 (27.27)	
When did you start wearing CA?	One year or less	11 (33.33)	22 (66.67)	0.005
	More than a year ago	23 (67.65)	11 (32.35)	
Do you like your smile?	Yes	31 (50)	31 (50)	0.667
	No	3 (60)	2 (40)	
BFI: extraversion	Score = 1:5	4 (30.77)	9 (69.23)	0.109
	Score = 6:10	30 (55.56)	24 (44.44)	
BFI: agreeableness	Score = 1:5	13 (56.52)	10 (43.48)	0.494
	Score = 6:10	21 (47.73)	23 (52.72)	
BFI: conscientiousness	Score = 1:5	5 (71.43)	2 (28.57)	0.247
	Score = 6:10	29 (48.33)	31 (51.66)	
BFI: neuroticism	Score = 1:5	14 (46.67)	16 (53.33)	0.548
	Score = 6:10	20 (54.05)	17 (45.95)	
BFI: openness to experience	Score = 1:5	5 (45.45)	6 (54.55)	0.701
	Score = 6:10	29 (51.79)	27 (48.21)	

There was no significant correlation between participants' satisfaction with their smiles and compliance levels. More importantly, personality traits did not show any statistically significant variations in compliance behavior for any BFI domains (p ≥ 0.05). Table 4 shows that a nonsignificant relationship was found between the instructions given to the participants and how frequently they attended follow-up visits (p ≥ 0.05).

**Table 4:** Correlation between compliance with instructions of attending appointments and BFI types BFI: Big Five Inventory

Variable	Score range	n	Compliant, n (%)	Not compliant, n (%)	p value
Extraversion	1:05	13	11 (84.6)	2 (15.4)	-
Extraversion	6:10	54	45 (83.3)	9 (16.7)	-
Agreeableness	1:05	33	21 (91.3)	2 (8.7)	0.307
Agreeableness	6:10	44	35 (79.5)	9 (20.5)	-
Conscientiousness	1:05	7	5 (71.4)	2 (28.6)	0.323
Conscientiousness	6:10	60	51 (85.0)	9 (15.0)	-
Neuroticism	1:05	30	23 (76.7)	7 (23.3)	0.199
Neuroticism	6:10	37	33 (89.2)	4 (10.8)	-
Openness to experience	1:05	11	8 (72.7)	3 (27.3)	0.371
Openness to experience	6:10	56	48 (85.7)	8 (14.3)	-

## Discussion

This study aimed to evaluate the impact of personality traits on compliance with CA therapy. However, our findings indicated no significant correlation between personality traits and compliance. This result contrasts with the findings of Xu and Tang [17], who reported a positive association between personality traits and adherence to clear retainers following fixed orthodontic treatment. Several factors may explain the nonsignificant result. First, the sample size (n = 67) may have been insufficient to detect subtle relationships between personality traits and compliance. Second, cultural differences between regions, such as Saudi Arabia versus China, could influence compliance behaviors in ways that are not captured by personality traits alone.

One of the challenges in this study was the reliance on self-reported compliance, which is prone to overestimation. Al-Moghrabi et al. [18] identified a discrepancy of 5.02 hours per day between self-reported and objectively measured wear time in patients using removable orthodontic appliances. Similarly, Schäfer et al. [19] emphasized that patients tend to overestimate compliance, suggesting that objective monitoring methods, such as clinical assessment, are essential for accurately assessing adherence in future research.

Our findings revealed that younger participants (aged 12-34) exhibited significantly higher compliance rates than older patients (p ≤ 0.05). This aligns with the findings of Schäfer et al. [19], which noted better adherence among younger individuals. The younger generations' higher motivation and adaptability to treatment requirements can explain this.

Furthermore, our study found that males demonstrated higher compliance than females (72.2% vs. 40.8%, p ≤ 0.05). This result, in line with Timm et al. [8], underscores the necessity of gender-specific strategies to enhance adherence in orthodontics. It highlights the importance of considering gender differences in treatment planning.

The results highlight that compliance decreases with longer treatment durations. Patients undergoing CA therapy for over a year showed significantly lower adherence than those being treated for one year or less (p ≤ 0.05). This is consistent with other studies [8,19], reflecting treatment fatigue as patients lose motivation over time. Developing strategies to maintain engagement throughout extended treatment may help mitigate this decline.

Contrary to expectations, satisfaction with one's smile was not significantly associated with compliance (p ≥ 0.05). This finding challenges the assumption that patients dissatisfied with their appearance are more motivated to adhere to treatment. Similar conclusions were drawn by Xu and Tang [17] and Pascoal et al. [15], suggesting that motivations beyond esthetics, such as functional improvements or social factors, may drive compliance behaviors.

This study is one of the first to explore personality traits as predictors of compliance in Saudi Arabia, which is a notable strength. By including diverse age groups, various clinics, and different personality types, the study enhances the generalizability of its findings. However, several limitations must be acknowledged. The sample size limited the ability to detect significant associations between personality traits and compliance, highlighting the need for future studies with larger and more diverse samples. The reliance on self-reported data may introduce biases, suggesting using objective monitoring tools in future research to improve accuracy. Additionally, examining the influence of cultural factors on compliance in the Middle East is crucial, as this area remains underresearched in orthodontics.

## Conclusions

Personality traits did not seem to have much of an impact on how well patients followed through with their CA treatment. However, factors like age, gender, and how long they had been in treatment were significant predictors of compliance. Patients who had been in treatment for less than a year were more likely to stick to it, and men tended to be more compliant than women. Younger patients, particularly those under 34, showed better adherence compared to older patients. Overall, compliance was moderate, with one of the key challenges being the hesitation to remove aligners in social settings, especially around meal times. Future research with larger, more diverse groups is needed to validate these results.

## Appendices

Appendix 1

**Table 5:** Compliance questions and scoring CA: clear aligner

Question	Answers	Score
Q1. Age (in years)	12:24	No score
	25:34	
	35 or more	
Q2. Gender	Male	No score
	Female	
Q3. Education level	Uneducated	No score
	Primary school	
	Middle school	
	High school	
	Bachelor	
	Master	
	Doctorate	
Q4. When did you start wearing orthodontic clear aligner treatment?	Less than a month	No score
	1-3 months	
	4-6 months	
	7-12 months	
	More than a year	
Q5. How often were you instructed to visit the doctor for follow-up?	1-2 months	Scoring is based on compliance
	3-6 months	Score = 3 if a + a, b + b, c + c, d + d, e + e
	7 months or more	Score = 2 if a + b, b + a, c + b, c + d, d + c, d + a
	When finished set	Score = 1 if a + c, b + c, b + d, a + d
	When you have a problem	Score = 0 if a + e, b + e, c + e, e + e
Q6. How often do you actually visit the doctor for follow-up?	1-2 months	No score
	3-6 months	
	7 months or more	
	When finished set	
	When there is a problem	
Q7. Do you wear your orthodontic clear aligner at work?	Always	4
	Most of the time	3
	Sometimes	2
	Rarely	1
	Never or not applicable	0
Q8. Do you wear your orthodontic clear aligner at school or college?	Always	4
	Most of the time	3
	Sometimes	2
	Rarely	1
	Never or not applicable	0
Q9. Do you wear your orthodontic clear aligner on weekends or off days?	Always	4
	Most of the time	3
	Sometimes	2
	Rarely	1
	Never or not applicable	0
Q10. Do you wear your clear aligner while sleeping?	Always	4
	Most of the time	3
	Sometimes	2
	Rarely	1
	Never	0
Q11. How many times do you remove your orthodontic clear aligner per day?	1-3 times	4
	4 times	3
	5 times	2
	6 times	1
	7 or more times	0
Q12. Why do you often remove your clear aligner? (select all that apply)	When I eat	No score
	When I drink	
	When I smoke	
	When I brush my teeth	
	When I talk to others	
	At work	
	At school or college	
Q13. How many hours per day does your doctor recommend you wear your CA?	22 hours or more	No score
	Most of the day	
	12 hours	
	Only while sleeping	
	No instructions given	
	Forgot instructions	
Q14. How many hours do you actually wear the aligner?	20-24 hours	4
	16-19 hours	3
	9-15 hours	2
	8 hours or less	1
	I don’t wear it	0
Q15. Do you wear the aligner for the exact hours recommended?	Yes	3
	No	0
Q16. Why are you not wearing the aligner as instructed? (select all that apply)	Don’t want to remove it to eat in front of people	No score
	Think wearing time is enough and effective	
	Affects speech	
	Doesn’t fit properly	
	Affects appearance	
	Pain and discomfort	
	Other	
Q17. Do you like your smile?	Yes	No score
	No	
Q18. Do you trust your doctor?	Yes	No score

Appendix 2

**Table 6:** BFI-10 questions BFI: Big Five Inventory

I see myself as someone who is	Strongly agree	Agree	Neither agree or disagree	Disagree	Strongly disagree
Discreet	-	-	-	-	-
Generally trusting	-	-	-	-	-
Tending to be lazy	-	-	-	-	-
Relaxed (handling stress well)	-	-	-	-	-
With artistic interests	-	-	-	-	-
Outgoing (sociable)	-	-	-	-	-
Finding faults in others	-	-	-	-	-
Doing a comprehensive job	-	-	-	-	-
Easily irritable	-	-	-	-	-
With an active imagination	-	-	-	-	-
